# Draft Genome Sequence of *Bacillus* sp. Strain EKM601B (Phylum *Firmicutes*), Living inside the Seeds of *Luffa acutangula* (Chinese Okra)

**DOI:** 10.1128/MRA.00180-20

**Published:** 2020-05-14

**Authors:** Eman M. Khalaf, Manish N. Raizada

**Affiliations:** a Department of Plant Agriculture, University of Guelph, Guelph, Ontario, Canada; b Department of Microbiology and Immunology, Faculty of Pharmacy, Damanhour University, Damanhour, Egypt; University of Arizona

## Abstract

Presented here is the draft genome sequence of *Bacillus* sp. strain EKM601B, which contains 4,199,360 bp in 73 contigs. This candidate endophyte was isolated from surface-sterilized dry seeds of *Luffa acutangula* (Chinese okra) and demonstrated diverse plant-beneficial functions and antagonism against soilborne pathogens *in vitro*.

## ANNOUNCEMENT

Seed-associated endophytic bacteria may be vectors for beneficial founder microbes that establish the plant microbiome (1). Many strains of the genus *Bacillus* are marketed as biofertilizers or biocontrol agents. They promote plant growth either directly via nutrient acquisition (2) or indirectly through antibiosis, competition, and induction of host defense responses (3). The seed microbiota of cucurbits (including cucumber, melons, pumpkin, squash, and luffa) were previously cultivated and extensively phenotyped, and *Bacillus* was the dominant genus (4, 5). The endophytic candidate *Bacillus* sp. strain EKM601B (GenBank accession number KT281323) was isolated from surface-sterilized seeds of *Luffa acutangula* (Chinese okra) in 2014 (4). The strain exhibited diverse *in vitro* growth-promoting functions, including growth on nitrogen-free medium and secretion of extracellular enzymes (pectinase, protease, and RNase) (4). Furthermore, this candidate endophyte displayed *in vitro* biocontrol activities via emission of volatile organic compounds (VOCs) (acetoin and diacetyl production) known to induce plant defenses and suppression of the soilborne fungal pathogen Rhizoctonia solani and the oomycete pathogen Phytophthora capsici (5).

Bacterial genomic DNA was isolated from an overnight LB broth culture (37°C, 250 rpm), which had been inoculated from a single colony streaked onto LB agar from the original glycerol stock, by using a DNeasy UltraClean microbial kit (Qiagen product number 12224-50) and then was adjusted to 50 ng/μl. DNA libraries were prepared using a TruSeq DNA Nano library preparation kit (KAPA HyperPrep kit, product number KK8504). The Illumina NovaSeq 6000 platform was used for sequencing, which generated 1,594,540 raw reads with an average length of 150 bp (paired end) with 97-fold coverage, compared to the top genome match in the database (Bacillus velezensis strain QST713; GenBank accession number CP025079.1) (6), as determined using KmerFinder v3.1 (7) with 99.85% query coverage. Using the EvoCAT (Evogene Clustering and Assembly Toolbox) pipeline, a total of 1,370,507 reads remained after trimming of low-quality sequences using a threshold score of 30. *De novo* assembly using EvoCAT resulted in 73 contigs (minimum scaffold length, 203 bp; maximum length, 644,951 bp; *N*_50_, 293,409 bp). The assembled genome is 4,199,360 bp, with a GC content of 47%. Prodigal software (8) was used for protein prediction; predicted proteins were subjected to searches against the NCBI nonredundant protein database using BLASTp (9). Protein domains were identified using InterProScan v5.32-71.0 software (10). Default parameters were used for all software unless otherwise specified.

The *Bacillus* sp. strain EKM601B genome is predicted to encode a minimum of 3,736 proteins. The genome annotation was consistent with the phenotypic traits expressed *in vitro* (4, 5), revealing candidate genes underlying the use of *Bacillus* species as biofertilizers and biocontrol agents (11–14). For example, data mining identified genes required for biological nitrogen fixation (*nifB* gene), secretion of extracellular enzymes that contribute to colonization or antimicrobial activity, such as pectin lyase and diverse proteases (e.g., serine proteases and metalloproteases), antimicrobial peptides (e.g., bacteriocin, which is reported to have broad-spectrum antimicrobial activity against R. solani and P. capsici [15]), ribonucleases (potential anti-RNA virus activity), butanediol-dehydrogenase-like (acetoin) production, and a wide variety of hydrolytic enzymes (in particular, chitinases [anti-insect and antifungal]). These preliminary findings may help to explain the success of *Bacillus* inoculants in agriculture.

### Data availability.

This whole-genome shotgun project has been deposited in DDBJ/EMBL/GenBank under the accession number JAALLJ000000000. The version described in this paper is the first version, JAALLJ010000000. Raw Illumina reads are available under SRA accession number SRR11051678.
